# Prevalence of autoimmune diseases in an admixed population of patients with type 1 diabetes: a multicenter study in Brazil

**DOI:** 10.1186/s13098-024-01274-3

**Published:** 2024-01-31

**Authors:** Marilia Brito Gomes, Fernanda Oliveira Braga, Karla Guerra Drummond, André Pinheiro, Franz Leal, Luís Cristóvão Porto, Livia Leite Ferreira, Geraldo da Rocha Castelar Pinheiro, Carlos Antonio Negrato

**Affiliations:** 1 Department of Internal Medicine, Diabetes Unit, State University of Rio de Janeiro, Boulevard 28 Setembro 77, Rio de Janeiro, 20551-030 Brazil; 2 Department of Ophthalmology, Sao Paulo Federal University, São Paulo. Av. Dr. Arnaldo, 455, Cerqueira César, São Paulo, SP Brazil; 3 Department of Ophthalmology, Regional Hospital of Taguatinga, QNC-Área Especial nº 24-Taguatinga Norte/DF, Brasília, Brazil; 4 Department of Ophthalmology, University of Campinas, Rua Tessália Vieira de Camargo, 126, Campinas, SP Brazil; 5 Histocompatibility and Cryopreservation Laboratory (HLA), State University of Rio de Janeiro (UERJ), Rio de Janeiro, Brazil; 6 Department of Internal Medicine, Reumathology Unit, State University of Rio de Janeiro, Rio de Janeiro, Brazil; 7 Medical Doctor Program, University of São Paulo-School of Dentistry, Bauru, São Paulo, Brazil

**Keywords:** Type 1 diabetes, Autoimmune diseases, Glycemic control, Diabetes-related chronic complications

## Abstract

**Background/objectives:**

The primary aim of this study was to evaluate the prevalence of autoimmune diseases (AIDs) and its associated factors in an admixed Brazilian population of patients with type 1 diabetes (T1D). The secondary one was to determine the relationship between AIDs and the occurrence of diabetes-related chronic complications (DRCC).

**Methods:**

This cross-sectional, nationwide survey was conducted in 13 public clinics in 11 Brazilian cities. Overall, 1,760 patients were included; 967 females (55.9%), 932 (54%) Caucasians, aged 29.9 ± 11.9 years, age at diagnosis 14.8 ± 8.9 years, diabetes duration 15.5 ± 9.3 years and 12.2 ± 3.8 years of school attendance. AIDs were retrieved from medical records or self-report and stratified as follows: absence of AIDs, only autoimmune thyroid disease (AITD), and other AIDs including the combination with AITD (hyper or hypothyroidism).

**Results:**

The prevalence of AIDs was 19.5% being AITDs (16.1%), the most frequently found. A higher prevalence of hypertension, dyslipidemia and overweight or obesity was found in patients who had exclusively AITDs. A higher prevalence of diabetic retinopathy (DR) was observed in patients with AITDs and patients with other AIDs in combination with AITDs. Chronic kidney disease (CKD) was more prevalent in patients with only AITDs. Lower levels of HbA1C, were observed in patients with isolated AITDs or with other AIDs, regardless of the presence of AITD. Hierarchical multivariate analysis, showed that AIDs were associated with female gender, older age, and longer diabetes duration, self-reported color-race (White and Brown), geographic region (Brazilian North/Northeast region) and higher anti-TPO levels (≥ 35 UI/ml).

**Conclusions:**

In conclusion, Brazilian patients with T1D, belonging to a highly ethnically admixed population, had an important prevalence of AIDs, mostly AITDs, that was associated with female gender, self-reported color-race, older age and longer diabetes duration. Moreover, these patients also had a higher prevalence of DRCC. Even though we highlight the importance of investigating the presence of AIDs at diagnosis and at regular intervals, it is unclear whether screening and early detection of additional AIDs may improve the clinical outcomes in individuals with T1D. Future prospective studies are necessary to establish the interplay between T1D, AIDs and DRCC.

**Supplementary Information:**

The online version contains supplementary material available at 10.1186/s13098-024-01274-3.

## Introduction

Type 1 diabetes (T1D) is an autoimmune disease (AID) and the most common endocrine condition found among children and adolescents worldwide. Its incidence is constantly increasing and it is estimated that more than 1.2 million children and adolescents had T1D in 2021 [1]. AIDs comprise a range of chronic diseases in which the immune response to self-antigens results in damage or dysfunction of the target organs. Patients with T1D present a much greater risk for developing other autoimmune conditions mainly autoimmune thyroid diseases (AITD) (hyper and hypothyroidism), celiac disease, skin diseases, and many others [2]. These diseases can occur sporadically or in combination, either with an insidious presentation or as an important hormonal decompensation [3].

Patients with an associated AID often present a deceleration in linear growth, overweight, delayed puberty (hypothyroidism), important variations in metabolic control (hyperthyroidism) and growth failure, poor weight gain, or weight loss (celiac disease) [4]. It should be noted that celiac disease is more frequently found in children with a younger age at diagnosis [5], while thyroid autoimmunity is more frequently found among children older than ten years [6]. The presence of these AIDs can negatively impact health, quality of life and cognitive function increasing the burden and the daily challenge to deal with T1D [5, 7].

The prevalence and types of combinations of AIDs with T1D vary widely depending on demographic variables such as gender, age, ethnicity, and diabetes duration [7–10]. In Finland, the country with the highest incidence of T1D, one in five patients with T1D has another associated AID [8]. In the USA, this association is found in one in four patients [9]. It should be mentioned that both studies were conducted mainly with Caucasian patients.

Data regarding minorities as well admixed populations with T1D are scanty. In the USA, the T1D Exchange Clinic Registry showed that Hispanic non-White and Black patients had lower frequencies of AIDs, of 21% and 12%, respectively, in contrast with a frequency of 29% found in White patients, corroborating the influence of ethnicity in autoimmune processes [3]. However, albeit the above-mentioned data, so far, the utility of screening patients with T1D for these diseases is not a consensus, mainly in older patients and those belonging to racial and ethnic minorities [3].

Another point that deserves attention is the possible relationship between different types of AIDs and diabetes-related chronic complications (DRCC). Few studies have addressed this subject [7, 9] and have used database from Catalonia in Spain [7] and from the USA [9]. Both studies have shown an association between the presence of AIDs with DRCC.

The primary aim of this study was to evaluate the prevalence of AIDs and its associated factors in an admixed Brazilian population of patients with T1D that participated in a multicenter study, the Brazilian Type 1 Diabetes Study Group (BrazDiab1SG). The secondary one was to determine the relationship between AIDs and the occurrence of DRCC**.**

## Patients and methods

This was a retrospective cross-sectional, multicenter study conducted between August 2011/August 2014 in 13 public clinics, located in 11 Brazilian cities, from all geographic regions. Research design and methods have been previously detailed [11]. Briefly, each clinic provided data from at least 50 outpatients with T1D receiving healthcare from the Brazilian National Health Care System (BNHCS). Our sample included 1,760 patients diagnosed with T1D between 1960 and 2014. This study was approved by the local ethics committee of each center. An endocrinologist followed all patients in these secondary or tertiary care centers. Patients were included if they were 13 years or older, had medical follow-up for at least six months at each respective center, and diagnosis of T1D made by a physician, based on the presence of classic clinical presentation at the moment of diagnosis, such as polyuria, weight loss, polydipsia, and the need for continuous insulin use since then. Patients who did not fulfill these criteria were excluded, as well as pregnant or lactating women and those who had an acute infection or ketoacidosis in the three months preceding the recruitment. Written informed consent was obtained from all patients and/or from their parents where necessary.

The following variables were assessed: current age, age at diagnosis, self-reported color-race (White, Black, Brown (“parda”), Asian (“amarela”) and Indigenous (“indígena”)) [12], diabetes duration, years of school attendance, smoking status, type of prescribed insulin therapeutic regimens (ITR) and body mass index (BMI). ITRs were stratified as follows: exclusive use of intermediate insulin (NPH) or long-acting insulin analogs, use of intermediate insulin (NPH) plus regular insulin or short acting insulin, use of long-acting insulin analogs plus short acting insulin, or use of continuous subcutaneous insulin infusion (CSII).

AIDs were retrieved from medical records or self-reported and stratified as follows: absence of AIDs, only AITD, and other AIDs, isolated or in combination with AITD. BMI was determined by dividing an individual’s weight (kg) by the square of the height (m^2^) [13]. Current smoking was defined as smoking more than one cigarette per day at the time of the interview. Hypertension in adults was self-reported. Patient’s awareness of hypertension was based on patient’s self-report of a prior hypertension diagnosis, made by a health care practitioner on at least two separate occasions. Regarding lipids, we considered the following values as normal: triglycerides < 150 mg/dL (1.7 mmol/L), high-density lipoprotein (HDL) cholesterol > 50 mg/dL (1.3 mmol/L) for women, and > 40 mg/dL for men (1.1 mmol/L) and low-density lipoprotein (LDL) cholesterol < 100 mg/dL (2.6 mmol/L) [14].

 HbA1c, creatinine, urea, triglycerides, total cholesterol, HDL cholesterol, LDL cholesterol, were measured using enzymatic techniques (BioSystem). Thyroid stimulating hormone (TSH), free levothyroxine (FT4), thyroid peroxidase antibodies (Anti-TPO) and vitamin B12 were measured by electrochemiluminescence (Cobas). HbA1c was measured using high-performance liquid chromatography (HPLC, Bio-Rad Laboratories, Hercules, California, USA). HbA1c at goal (good glycemic control) was defined as HbA1c < 7.0% (53 mmol/mol) [14]. Inadequate glycemic control was defined as HbA1c ≥ 7.0% (53 mmol/mol). Serum uric acid was measured using an uricase-based commercial kit (BioSystem) with results expressed in milligrams per deciliter (mg/dL) and normal range between 3.5–7.2 mg/dL for men and 2.6–6.0 mg/dL for women. Friedewald’s equation was used to calculate LDL cholesterol values [15]. Creatinine was measured using a colorimetric assay kit and C-reactive protein (CRP) was measured by immunoturbidimetry (Biosystems). All the above laboratorial data including HbA1c levels were measured in a single center (State University of Rio de Janeiro).

In adolescents, normal weight was defined as a BMI ≥ 3th and ≤ 85th percentile, underweight as a BMI < 3rd percentile, overweight as a BMI > 85th percentile, and obesity as a BMI > 97th percentile according to age and gender [13]; hypertension was defined as systolic blood pressure and/or diastolic blood pressure ≥ 95th percentile for age, sex and height or 130 × 80 mmHg to 139 × 89 mmHg for children aged 10 to < 13 years and ≥ 130 × 80 mmHg for those with ≥ 13 years old [16]

### Evaluation of renal function and retinopathy

Renal function was estimated by the CKD-EPI equation [17] in adults and by the Schwartz formula in adolescents [18] and was expressed as glomerular filtration rate (eGFR) in milliliters per minute per 1.73 m^2^ (ml/min). We considered all patients as non-African-Americans in the CKD-EPI equation. Albuminuria concentration was measured from a morning urine sample. Patients were instructed to avoid physical activity before collecting the urine sample. Patients submitted to kidney transplantation and those with urinary infection or hematuria were excluded. This procedure was repeated twice with a minimal interval of one week and maximal of six months between each collected sample. Urinary albumin was evaluated by immunoturbidimetry (method detection limit: 0.09 mg/dL). The presence of albuminuria was defined as albuminuria ≥ 30 mg/dL. The dosage of albuminuria was considered in those patients that collected at least two urine samples, and the mean of these samples classified the patients as having albuminuria or not.

Patients were divided into two groups, as having: normal renal function or CKD. Patients with normal renal function had a GFR ≥ 60 ml/min and the absence of albuminuria. CKD was defined as a GFR < 60 mL/min and/or the presence of albuminuria [19].

The screening for diabetic retinopathy (DR) was performed by mydriatic binocular indirect ophthalmoscopy (BIO), which was performed by an experienced retinal specialist in each center, who was trained before the beginning of the study in an ophthalmologic University center. The classification of DR for each patient was assessed for the eye with the highest level of commitment. Each eye was classified based on the absence or presence of DR, that was classified as: absent, non-proliferative DR (NPDR), proliferative DR (PDR) and macular edema, according to the international classification of DR [20]

### Sample calculation, economic status

A detailed description of the study sample calculation has been described previously [21]. Briefly, the study sample represented the distribution of T1D cases across four geographic regions of Brazil. This distribution was estimated using the overall population distribution reported in the 2000 Brazilian Institute of Geography and Statistics Population Census (IBGE) [22]. These data were combined with national estimates of diabetes prevalence derived from a 1988 survey to determine the minimum number of patients to be studied in each region [23]. Economic status was defined according to the Brazilian Economic Classification Criteria [24]. The following economic status categories were considered for this analysis: high, middle, low and very low. This classification also accounts for education level: illiterate/incomplete primary education, complete primary education/incomplete secondary education, complete secondary education/incomplete high school, complete high school/some college, or college graduate [25].

### Statistical analysis

Firstly, an exploratory analysis was performed to assess clinical, demographic and laboratory data stratified according to the presence of AIDs as follows: absence of AIDs, presence of only AITD and other AID alone or combined with AITD. These data were presented as means (± SD) or median, interquartile range [IQR] or minimum and maximum for continuous variables and as counts (relative frequencies) for discrete variables. For categorical variables analysis, including the presence of DR (yes/no) and CKD (yes/no), the Chi-square test was used. For continuous variables we have used Anova with Sidak correction for multiple comparisons and Student T test.

 As we did not observe any relevant difference between the group with only AITD and the group with other AID alone or combined with AITD, both were combined for multivariate analysis. Hierarchical multivariate logistic analysis with the presence of AIDs (yes/no) as the dependent variable was performed in two levels: Sociodemographic status (Model 1) and laboratorial data (Model 2). In Model 1 as independent variables, we considered all those with statistical significance in the exploratory analysis (p < 0.2) or with clinical relevance found in the literature: age, gender, self-reported color-race (White, Black, Brown; as Asians or Indigenous comprised only 2% of our sample they were excluded), geographic regions, economic status, time of follow-up in each center, level of care and diabetes duration. In model 2 (Laboratorial data) CRP levels and anti-TPO (≥ 35 UI/mL) were the independent variables. Model fit was assessed through Hosmer and Lemeshow and Omnibus test. Nagelkerke R^2^ was calculated and Odds ratio (OR) with 95% confidence interval (CI) were expressed where indicated. Sidak correction was applied when ANOVA test was used. All analyses were performed using the Statistical Package for the Social Sciences (SPSS version 17.0, SPSS, Inc., Chicago, Illinois, USA). A two-sided *p* value less than 0.05 was considered significant.

## Results

### Overview of the prevalence of AIDs and the socio demographic data of the studied population

Overall, AIDs were found in 344 patients (19.5%). The majority of them presented autoimmune hypothyroidism, n = 258 (14.7%), followed by hyperthyroidism, n = 26 (1.5%). Overall, 18 patients (1.0%) presented a combination of AIDs being the most frequent the combination of AITD with vitiligo (n = 6 patients) and with rheumatoid arthritis (n = 6 patients). Sociodemographic data of the studied population are listed in Table 1.

**Table 1:** Sociodemographic data of the studied population

Age, y	30.2 ± 11
Gender, F (%)	987 (56.1%)
Diabetes duration, y	15.5 ± 9.3
Self-reported color-race, n (%)	
White	958 (54.4%)
Black	136 (7.7%)
Brown	631 (35.9)
Asian	19 (1.1)
Indigenous	16 (0.9)
Years of school attendance, y	12.2 ± 3.8
Economic status	
High	53 (3%)
Medium	801 (45.5%)
Low	849 (48.2%)
Very low	57 (3.2%)
Geographic region (%)	
Southeast	829 (47.1%)
South	233 (13.2%)
North and Northeast	490 (27.8%)
Midwest	208 (11.8%)
Level of care, n (%)	
Secondary	651 (37%)
Tertiary	1109 (63%)
Physical activity	
Yes	910 (51.7%)
No	848 (48.2%)
Smoking	
Yes	227 (13.8%)
No	1414 (86.2%)

Data are presented as the means (SD) and n (%). y, year; F, female

**Fig. 1:**
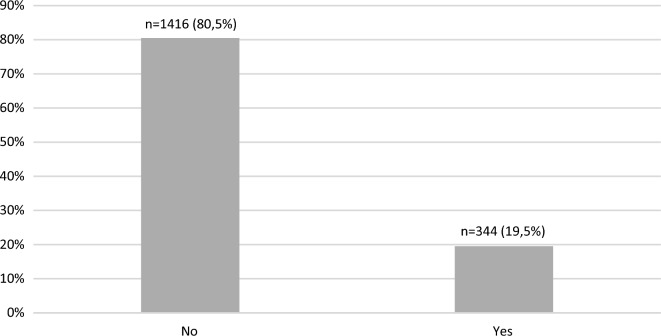
Prevalence of autoimmune diseases in the studied population

**Fig. 2:**
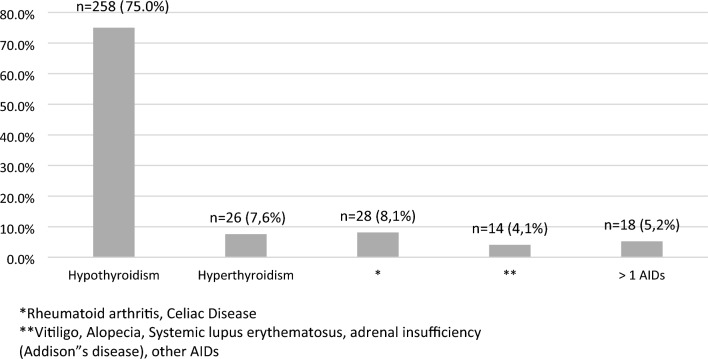
Type of autoimmune diseases in patients with AIDs (n = 344)

The overall prevalence of AIDs and its distribution are depicted in Figs. 1, 2 and 3, respectively.

### Prevalence of AIDs and its associated factors 

The overall prevalence of AIDs was 19.5% (n = 344). Considering the exploratory analysis, a higher prevalence of exclusively AITD n = 284 (16.1%) and of other AIDs regardless of the presence of AITDs n = 60 (3.4%) was observed in female patients in comparison to male patients, [n = 206 (72.5%) vs n = 38 (63.5%) vs n = 745 (52.6%)] vs [n = 78 (27.5%) vs n = 22 (36.7%) vs n = 671 (47.4%)], respectively, p < 0.001); a higher prevalence of exclusively AITD was observed in White patients in comparison to Brown and Black patients, [n = 183 (64.4%) vs n = 87 (30.6%) vs n = 7 (2.5%)], respectively, p < 0.0001) and a higher prevalence of other AIDs regardless of the presence of AITDs was observed in White patients in comparison to Brown and Black patients n = 42 (70.0%) vs n = 16 (26.7%) vs n = 2 (3.3%), respectively, p < 0.0001). Patients with exclusively AITDs and other AIDs regardless of the presence of AITDs had longer diabetes duration in comparison to patients without any AIDs [(18.2 ± 10.5 vs 19.6 ± 9.3 years vs 14.8 ± 8.6)], respectively, p < 0.001. Patients with exclusively AITDs and other AIDs regardless of the presence of AITDs were older in comparison to patients without any AIDs [(33.8 ± 12.9 vs 37.3 ± 11.9 vs 28.9 ± 11.4], respectively, p < 0.001. A higher prevalence of hypertension, dyslipidemia and overweight/obesity was observed in patients with exclusively AITDs in comparison to patients without any AIDs [75(26,4%) vs 226 (16.0%)], p < 0.001; [86 (30.4% vs 272(19.3%), p < 0.001)] and [125 (44.0%) vs 504 (36.7%), respectively, p = 0.009].

A higher prevalence of DR was observed in patients with exclusively AITD and with other AIDs independent of the presence of AITDs in comparison to patients without any AIDs, [118 (42.1%) vs 469 (34.1%), p = 0.01] and [30 (49.2%) vs 469 (34.1%), respectively, p = 0.02]. A higher prevalence of CKD was observed in patients with exclusively AITDs in comparison to patients without any AIDs [(338(33.2%) vs 88(43.1%), respectively, p = 0.008]. Patients with exclusively AITDs and other AIDs regardless of the presence of AITDs had lower rates of GFR (mL/min/1.73m^2)^) in comparison to patients without any AIDs [(78.4 ± 28.0 vs 79.4 ± 21.0 vs 89.2 ± 30.5 ml/min), respectively, p < 0.05]. Patients with exclusively AITDs and other AIDs regardless of the presence of AITDs had higher frequency of anti-TPO above 35UI/ml, than patients without any AIDs, [(173 (65.3) vs 18 (32.7) vs 227 (16.8), respectively, < 0.001].

Patients with exclusively AITD and with other AIDs independent of the presence of AITD in comparison with patients without any AID had lower HbA1c levels [(8.7 ± 1.9% vs 8.6 ± 2.1% vs 9.1 ± 2.1),] p = 0.006, respectively). These data are described in Table 2.

**Table 2:** Clinical, demographic and laboratory data stratified by the presence of autoimmune disease (AIDs)

	Autoimmune diseases n (%)			
	No 1416 (80.5)	Only thyroid AID 284 (16.1)	Other AID ^$^ 60 (3.4)	*p-value
*Demographic data*				
Gender				
Female, n (%)	745 (52.5)	206 (72.5)	38 (63.5)	< 0.001
Male,n(%)	671(27.5)	78(27.5)	22(36.7)	
Age, y	28.9 ± 11.4	33.8 ± 12.9	37.3 ± 11.9	< 0.001
< 19	329 (27.9)	34 (17.1)	4 (6.7)	
≥ 19 to < 30	551 (38.9)	101 (35.6)	16 (26.7)	
≥ 30 to < 40	298 (21.0)	64 (22.5)	14 (23.3)	
≥ 40	238 (16.8)	85 (29.9)	26 (43.3)	
Diabetes duration, y	14.8 ± 8.6	18.2 ± 10.5	19.6 ± 9.3	< 0.0001
< 5	108 (7.6)	12 (4.2)	1 (1.7)	
≥ 5 to < 10	402 (28.4)	59 (20.8	9 (15.0)	
≥ 10 to < 20	587 (41.5)	110 (38.7)	23 (38.3)	
≥ 20	319 (22.5)	103 (36.3)	27 (45.0)	
Age at diagnosis, y	14.4 ± 8.6	15.6 ± 10.0	17.1 ± 9.5	0.009
< 12	703 (49.6)	128 (45.1)	18 (30.0)	
≥ 12 to < 19	385 (27.2)	73 (25.7)	19 (31.7)	
≥ 19	328 (23.2)	89 (29.2)	23 (38.7)	
Time of follow-up, y	9.3 ± 7.8	10.3 ± 8.5	13.4 ± 8.6	< 0.001
Tertiary level of care, n (%)	863(60.9)	200(70.4)	46(76.7)	0.001
Self-reported color-race, n (%)				< 0.001
White	735(51.8)	183(64.4)	42(70.0)	
Black	127 (9.0)	7(2.5)	2(3.3)	
Brown	528(37,3)	87(30.6)	16(26.7)	
Asian	14(1.0)	5(1.8)	0	
Indigenous	14(1.0)	2(0.7)	0	
Geographic region, n (%)				< 0.001
Southeast	645(45.6)	148(52.1)	36(60.0)	
South	172(12.1)	53(18.7)	8(13.3)	
North/Northeast	431(30.4)	51(18.0)	8(13.3)	
Mid-west	168(11.9)	32(11.3)	8(13.3)	
Economic status (%)				0.13
High	41(2.9)	10(3.5)	2(3.3)	
Medium	630(44.5)	142(50.0)	29 (48.3)	
Low	693(48.9)	130(45.8)	26(43.3)	
Very low	52(3.7)	2(0.7)	3(5.0)	
Years of study, y	12.2 ± 3.8	12.0 ± 3.7	12.9 ± 4.1	0.24
Smoker, yes n (%)	69(4.9)	19(6.7)	4(6.8)	0.4
Diabetes management and treatment				
Number of clinical visits per year	3.6 ± 1.7	3.8 ± 1.8	4.2 ± 1.8	0.009
NPH or long acting analogs^**†**^	77 (5.4)	7 (2.5)	2 (3.3)	0.026
NPH + regular or analogs/ Insulin analogs (long + short acting)	1297 (91.6)	260 (91.5)	55 (91.7)	
Continuous subcutaneous insulin infusion	42 (3.0)	17 (6.0)	3 (5.0)	
Clinical data and comorbidities				
BMI, kg/m^2^	24.0 ± 4.1	24.9 ± 4.3	23.8 ± 4.1	0.003
Hypertension, yes n (%) ***	226 (16.0)	75 (26.4)	11 (18.3)	< 0.001
Dyslipidemia, yes n (%)	272 (19.3)	86 (30.4)	17 (28.8)	< 0.001
Overweight or obesity	504 (36.7)	124 (44.0)	19 (31.7)	0.02
Laboratorial data				
HbA1c (%)	9.1 ± 2.1	8.7 ± 1.9	8.6 ± 2.1	0.006
HbA1c (mmol/mol)	75.8 ± 23.6	71.5 ± 21.1	71.0 ± 23.2	
TSH (mUI/L)	2.1 (1.6)	2.9 (3.7)	2.0 (1.6)	< 0.001
FT4 (ng/dL)	1.26 ± 0.26	1.4 ± 0.4	1.27 ± 0.26	< 0.001
Anti TPO > 35 (UI/mL)	227 (16.8)	173 (65.3)	18 (32.7)	< 0.001
Anti TPO (UI/ml)	14.5[14.7]	131.5[311.8]	15.3[92.8]	< 0.001
B12 vitamine (pg/ml)	557.2 ± 243.1	517.8 ± 278.7	475.5 ± 192.7	0.005
C reactive protein (mg/dL)	0.16[0.41]	0.22[0.56]	0.19[0.45]	0.12
Medications				
Anti-hypertensive drugs, yes n (%)	363 (25.8)	93 (33.2)	22 (36.7)	0.01
Statin, yes n (%)	260 (18,4)	98 (34.5)	24 (40.0)	< 0.001
Thyroid hormone	35 (2.5)	261 (91.9)	16 (26.7)	< 0.001
Diabetes-related chronic complications				
Retinopathy, yes n (%)	469 (34.1)	118 (42.1)	30 (49.2)	0.004
CKD, yes n (%)^**†††**^	338 (33.2)	88 (43.1)	16 (37.2)	0.02
GFR, mL/min/1.73m^2**††††**^	89.2 ± 30.5	78.4 ± 28.0	79.4 ± 21.0	< 0.001
Albuminuria, mg/dL	9.4 (18.7)	8.4 (19.8)	7.5 (20.4)	0.59

Data are presented as n (%), mean ± SD or median [IQR, interquartile range]; ^$^ other AIDs (regardless the presence of thyroid AID) * p < 0.05 was considered significant.; *** Hypertension was defined as elevated blood pressure (systolic or diastolic) ≥ 130 × 85 mmHg or the use of antihypertensive drugs); ^**†††**^ CKD: chronic kidney disease; ^**††††**^ GFR: glomerular filtration rate

### Hierarchical multivariate logistic analysis with AIDs as the dependent variable

Hierarchical multivariate analysis performed with AIDs (yes/ no) as the dependent variable, showed that all the independent variables that entered the model, even after adjustment, could explain only 29.9% (Nagelkerke R-squared) of a given patient having AIDs. Overall, 83.3% of these patients were correctly classified after the adjustment of Model1 and Model 2. AIDs were associated with female gender, older age, longer diabetes duration, self-reported color-race (White and Brown), geographic region (lower OR for Brazilian North/Northeast region) and anti-TPO levels ≥ 35 UI/mL. The final adjusted model is described in Table 3.

**Table 3:** Hierarchical multiple logistic regression analysis of sociodemographic and laboratorial data associated with the presence of autoimmune diseases

Variables	Adjusted OR	95%CI	P^*^
*Sociodemographic level (Model 1)*			
Female (Ref.: Male)	1.713	1.270–2.311	< 0.001
Age, years (Ref.: < 19)			
19 up to < 30	2.063	1.233–3.53	0.006
≥ 30 to < 40	2.368	1.349–4.158	0.003
≥ 40	3.534	1.953–6.394	< 0.001
Self-reported skin-color (Ref.: Black)			
White	4.188	2.077–8.444	< 0.001
Brown	3.018	1.474–6.178	0.003
Level of care (Ref.: Secondary)			
Tertiary	1.141	0.741–1.757	0.548
Diabetes duration, years	1.018	1.000–1.036	0.047
Time of follow up, years	0.996	0.976–1.016	0.662
Geographic region (Ref. Mid-West)			
Southeast	1.071	0.596–1.925	0.818
South	1.076	0.537–2.156	0837
North/Northeast	0.494	0.288–0.849	0.01
Economic status (Ref. High)			
Medium	0.803	0.359–1.798	0.594
Low	0.678	0.301–1.523	0.346
Very low	0.464	0.117–1.838	0.274
*Laboratorial data (Model 2)*			
C-reactive protein (mg/dl)	0.914	0.779–1.073	0.545
Anti-TPO UI/ml^**^ (Ref. < 35)			
≥ 35	7.703	5.756–10.309	< 0.001

*OR*  odds ratio, *95% CI*  95% confidence interval, *Ref.*  Reference category. *P < 0.05. Model 1: Odds ratio adjusted for factors from Model 1. Model 2: Odds ratio adjusted by factors from Models 1 and 2**. *Anti-TPO* thyroid peroxidase antibodies

## Discussion

The present study, found an overall prevalence of AIDs of 19.5% in Brazilian patients with T1D belonging to a highly admixed population. The majority of patients presented AITD, being hypothyroidism the alteration most frequently found, followed by hyperthyroidism. Overall, 18 patients presented a combination of AIDs, being the association of AITD with vitiligo and with rheumatoid arthritis the most prevalent.

The presence of AIDs was associated with female gender, older age, self-reported color-race (White and Brown), longer diabetes duration, region of the country (North/Northeast) and anti-TPO ≥ 35 UI/mL. A higher prevalence of DR and lower levels of HbA1C were observed in patients with AITDs and patients with other AIDs in combination with AITDs. CKD was more prevalent in patients with only AITDs.

Differences in the prevalence of AIDs in patients with T1D have been described worldwide and have been related to methodological, demographic and ethnic factors [7–10]. In general, most of these studies have shown a prevalence ranging from 18.3 to 27% being AITD the most common, similar to our findings.

We have found a higher prevalence of AIDs either exclusively AITD or AITD combined with other AIDs in women. The higher prevalence of AIDs in women with T1D is a universal finding although AIDs can also be found in men in up 30% [10] similar to our findings (~ 28%). These data have also been described in a national data base from the USA [26], in which, a heterogeneity regarding geographic areas and ethnicity was also found. Ethnicity is an important factor in Brazil, a country with an important racial diversity and a heterogeneous ancestry, since it was originally formed by three ancestral roots that are highly admixed: Native Americans, Europeans, and Sub-Saharan Africans [27]. Miscegenation among these 3 roots has occurred over centuries, resulting from asymmetric mating patterns, mainly between European men and Native American or African women [27]. Since 1991, self-reported race/skin color has been used for Brazilian population censuses, however, this can lead to misclassification of some individuals [12] as has been demonstrated in a national T1D multicenter study [28]. In this study, those patients who self-reported as being White and Brown had a greater percentage of European genomic ancestry than Black patients reflecting the history of our immigration [28]. Meanwhile, self-reported Black patients still present European genomic ancestry but at a lower proportion. Although this fact could have influenced our results, this study has shown a gradient of AIDs prevalence, from White to Brown and Black patients. A North American study carried out with an admixed population of T1D, the T1D Exchange Clinic Registry that showed that Hispanic Non-White and Black patients had a lower frequency of AIDs, of 21% and 12%, respectively, in contrast with a 29% frequency found in White patients [3]. In the present study, patients form North /Northeast region had a decrease OR for the presence of AIDS. This fact may be associated with a lower frequency of individuals who declare themselves as being White, as well as a lower frequency of Caucasian genomic ancestry in patients with T1D in this region as previously described [28]. It is important to note that patients who self-reported as being Black or Indigineous had lower prevalence of AITDs and AITDS in combination with other AIDs showing the importance of specifying different ethnic groups in populational studies. This latter fact had been shown in a previous Brazilian study conducted in the Northeast region that classified patients as being White and Non-White, and has found no difference between the prevalence of AITDs between the two groups [29].

Similar to our data, a comparative study conducted with the general population in USA [26], evaluating 22 AIDs rates by sex, geographic region, and race has found a considerable heterogeneity across geographic regions and racial groups, showing that potential variation factors may include genetic susceptibility and/or environmental factors. The evaluated AIDs were: acquired hemophilia A, alopecia areata, autoimmune hemolytic anemia, autoimmune hypoparathyroidism, autoimmune neutropenia, chronic inflammatory demyelinating polyneuropathy, dermatitis herpetiformis, Guillain–Barre syndrome, immune thrombocytopenic purpura, myasthenia gravis, polymyositis/dermatomyositis, primary biliary cirrhosis, scleroderma, Sjögren’s syndrome, celiac disease, Addison’s disease, multiple sclerosis, ulcerative colitis, T1D, rheumatoid arthritis, Crohn’s disease and systemic lupus erythematosus [26].

In the present study, that evaluated only patients with T1D, it was found lower rates of AIDs than this previous American study, that evaluated the general population [26]. The AIDs found either isolated or in combination with AITD were rheumatoid arthritis, celiac disease, vitiligo, alopecia, systemic lupus erythematosus and Addison’s disease.

However the prevalence of these AIDS was similar to the prevalence described in another study [30]. This finding could be due to the fact that screening for AIDs in the BNHCS is not satisfactory, or that by having an insidious presentation AIDs are difficult to be diagnosed and finally to the genetic profile of the highly admixed Brazilian population.

The diagnosis of AITD was made by the presence of thyroid-specific autoantibodies, mostly thyroid peroxidase antibodies (anti-TPO) in serum, and by varying degrees of thyroid dysfunction [31]. We have observed a prevalence of anti-TPO of 25% similar to what was found in another Brazilian study which comprised only young patients with T1D.

We observed that older age and longer diabetes duration have been associated with the presence of AIDs as noted in other studies [7–10, 29–33]. However, it is important to highlight that some AIDs like celiac disease, are frequently observed in children and adolescents [3]. In the T1D Exchange Clinic Registry it was observed that the frequency of two or more AIDs increased from 4.3% in patients younger than 13 years to 10.4% in those older than 50 years [3]. Also, a metanalysis performed to estimate the pooled prevalence of AIDs in patients with T1D, has shown that for every 10-year age increase, the prevalence of hypothyroidism increased 4.6% [32].

Data are still controversial regarding the relationship between the presence of AIDs and the occurrence of DRCC [7–9]. We noted, a higher prevalence of DR and CKD in patients with AIDs. This is in accordance with the findings of a longitudinal study conducted in the USA that has found that patients with T1D, mainly women, frequently present the coexistence of AIDs that are associated with higher rates of renal failure, ischemic stroke, and myocardial infarction [9]. However, in Catalonia, patients who presented only AITD had lower prevalence of kidney disease and peripheral artery disease. In contrast, those patients with other AID, except AITD had a higher prevalence of DR, neuropathy, ischemic heart disease and cerebrovascular disease [7].

The present study also observed a higher prevalence of hypertension, dyslipidemia and overweight or obesity in patients with AITD alone. Patients with T1D from the Catalonian study with only AITDs or with any AIDs used more statins and anti-hypertensive drugs, which could be related to treatment of dyslipidemia and hypertension, respectively [7]. Although a difference in HbA1c levels was observed among the groups, no difference in the proportion of patients who reached good glycemic control was observed, similar to data described in the Catalonian study [7].

In the present study it was noted a difference in the prevalence of AIDs among different regions of the country, being the Northeast, the region with the lowest rates. This fact has also been noted in Europe concerning the prevalence of hypothyroidism that was found to be higher in southeast [32]. So far, there is no explanation for this finding, but we can speculate that it could be related to ethnicity and genomic ancestry (Caucasian) as we have discussed above, and genetic predisposition, mainly related to the frequency of the alleles of the histocompatibility leukocyte antigen system (HLA), located on chromosome 6p21 and involved in human immune response [2]. For instance, the combination of alleles of the HLA system (haplotypes) such as DRB1*03:01; DQA1*05:01; DQB1*02:01 and DRB1*04(01;02;04;05); DQA1*03:01 and DQB1*03:02 are the common genetic background for many AIDs [33, 34]. These haplotypes have conferred an increased risk for T1D in a Brazilian cohort and were more common in patients who self-reported as being White and with higher percentage of Caucasian genomic ancestry [35, 36].

The alleles DRB1*03:01 or DRB1*04:(01;02;04;05) have also been associated with some AIDs like celiac disease, AITD, adrenal insufficiency, autoimmune gastritis and reumathoid artritis [32]. All these aforementioned AIDs are the most usually found in patients with T1D as pointed out in a recent review as well as in the present study [32], corroborating the hypothesis that they share commom pathogenetic mechanisms. However we can not rule out the interplay between genetic predisposition and enviromental factors as well the participation of other genes outside the HLA region [33, 34]. Recently the participation of the dysbiosis in this pathogenic process has been pointed out [37].

The main strength of our sample is that it represents the diverse, young Brazilian population with T1D, with a diverse multiethnic and socioeconomic background, belonging to all geographic regions of the country. Also, a uniform and standardized recruitment protocol in all participating centers was used.

Finally, our study has also some limitations that must be addressed. The first was the sample characteristics. All patients lived in large cities and received medical care in public health care centers by a specialist; thus, patients who rely on primary care facilities and live in rural areas may not have been represented. However, the former patients with T1D are the minority of those receiving treatment in Brazil. Second, the stratification of AIDs could be considered a limitation since it did not allow us a better discrimination of each one. However, this stratification was previously described in a Spanish study [7]. Third, our study had a cross-sectional design that does not allow us to establish a causality association between the presence of AIDs and DRCC. Fourth, as we did not have the timing of onset of each AID, the time frame between T1D and the presence of other AID could not be established.

In conclusion, our study showed that patients with T1D, belonging to a highly ethnically admixed population, had an important prevalence of AIDs, mostly AITD, that was associated with female gender, self-reported color-race, older age and longer diabetes duration. Moreover, these patients also had a higher prevalence of DRCC. Even though we highlight the importance of investigating the presence of AIDs at diagnosis and at regular intervals, it is unclear whether screening and early detection of additional AIDs may improve the clinical outcomes in individuals with T1D. Future prospective studies are necessary to establish the interplay between T1D, AIDs and DRCC.

### Supplementary Information

**Additional file 1: Table S1.** Brazilian Type 1 Diabetes Study Group (BrazDiab1SG).

## Data Availability

The used datasets and/or analyzed during the current study are available with the corresponding author upon reasonable request.

## Abbreviations

AIDs: Autoimmune diseases
T1D: Type 1 diabetes
AITD: Autoimmune thyroid disease
BrazDiab1SG: Brazilian Type 1 Diabetes Study Group
BMI: Body mass index
BNHCS: Brazilian National Health Care System
HbA_1_C: Glycated hemoglobin
CSII: Continuous subcutaneous insulin infusion
DR: Diabetic retinopathy
NPDR: Non-proliferative diabetic retinopathy
PDR: Proliferative diabetic retinopathy
ITR: Insulin therapeutic regimen
eGFR: Estimated glomerular filtration rate
CRP: C-reactive protein
TSH: Thyroid stimulating hormone
FT4: Free levothyroxine
Anti-TPO: Thyroid peroxidase antibodies
HLA: Histocompatibility leukocyte antigen system
