# Supplementary material for: Prevalence of autoimmune diseases in an admixed population of patients with type 1 diabetes: a multicenter study in Brazil
Source: Diabetol Metab Syndr. 2024 Jan 31;16:31. doi: 10.1186/s13098-024-01274-3 (PMC10829295; doi:10.1186/s13098-024-01274-3)
Supplement: Supplementary file 1 — Additional file 1: Table S1. Brazilian Type 1 Diabetes Study Group (BrazDiab1SG). [file 13098_2024_1274_MOESM1_ESM.docx]

**Additional**

**Table S1: Brazilian Type 1 Diabetes Study Group (BrazDiab1SG)**

Executive steering committee: Marilia Brito Gomes (chair), Carlos Antonio Negrato.

Participants and principal investigators( indicated by an asterisk).

| Marilia Brito Gomes* | State University of Rio de Janeiro | [mariliabgomes@gmail.com](mailto:mariliabgomes@gmail.com) |
| --- | --- | --- |
| Laura Nunes Melo | State University of Rio de Janeiro | [lauragnmelo@gmail.com](mailto:lauragnmelo@gmail.com) |
| Alessandra Matheus | State University of Rio de Janeiro | [alessandramatheus79@yahoo.com](mailto:alessandramatheus79@yahoo.com) |
| Roberta Cobas | State University of Rio de Janeiro | robertacobas@gmail.com |
| Lucianne Righeti Monteiro Tannus | State University of Rio de Janeiro | luciannetannus@ig.com.br |
| Melanie Rodacki* | Federal University Hospital of Rio de Janeiro | mrodacki2001@yahoo.com.br |
| Lenita Zajdenverg | Federal University Hospital of Rio de Janeiro | lenitazaj@gmail.com |
| Joana Rodrigues Dantas | Federal University Hospital of Rio de Janeiro | joanardantasp@ig.com.br |
| Maria Lúcia Cardillo Corrêa-Giannella* | University Hospital of São Paulo | malugia@lim25fm.usp.br |
| Sharon Nina Admoni | University Hospital of São Paulo | sharonadmoni@ gmail.com |
| Daniele Pereira dos Santos | University Hospital of São Paulo | dps.daniele@ hotmail.com |
| Carlos Antonio Negrato* | Bauru’s Diabetics Association | carlosnegrato@uol.com.br |
| Maria de Fatima Guedes | Bauru’s Diabetics Association | tatiguedeses@hotmail.com |
| Sergio Atala Dib* | Federal University of São Paulo State | sergio.dib@unifesp.br |
| Celso Ferreira de Camargo Sallum Filho | Federal University of São Paulo State | celsosallum@superig.com.br |
| Paulo Henrique Morales | Federal University of São Paulo State | phmorales@institutodavisao.org.br |
| Fernando Malerbi | Federal University of São Paulo State | fernandokmalerbi@gmail.com |
| Karla Guerra Drumond | Federal University of São Paulo State | guerradrummond@gmail.com |
| Elisabeth João Pavin* | University of Campinas | [ejpavin@fcm.unicamp.br](mailto:ejpavin@fcm.unicamp.br) |
| Franz Schubert Leal | University of Campinas | franzschubertleal@gmail.com |
| Caroline Takano | University of Campinas | caroline.takano@gmail.com |
| Rosângela Roginski Rea* | Federal University of Paraná | rosangelarea@uol.com.br |
| Nicole Balster Romanzini | Federal University of Paraná | nikbr@hotmail.com |
| Mirela Azevedo* | Clinical Hospital of Porto Alegre | mirelajobimazevedo@gmail.com |
| Luis Henrique Canani | Clinical Hospital of Porto Alegre | luishenriquecanani@gmail.com |
| Felipe Mallmann | Clinical Hospital of Porto Alegre | felipekmallmann@gmail.com |
| Hermelinda Cordeiro Pedrosa* | Regional Hospital of Taguatinga | pedrosa.hc@globo.com |
| Monica Tolentino | Regional Hospital of Taguatinga | monicatolentino@uol.com.br |
| Cejana Hamu Aguiar | Regional Hospital of Taguatinga | cejanahamu@yahoo.com.br |
| André Pinheiro | Regional Hospital of Taguatinga | andrepip@gmail.com |
| Reine Marie Chaves Fonseca* | Diabetes and Endocrinology Center of Bahia | reinemar@terra.com.br |
| Ludmila Chaves Fonseca | Diabetes and Endocrinology Center of Bahia | ludchaves@yahoo.com.br |
| Tessa Mattos | Diabetes and Endocrinology Center of Bahia | tessamattos@gmail.com |
| Raffaele Kasprowicz | Diabetes and Endocrinology Center of Bahia | raffaellebarros@hotmail.com |
| Adriana Costa e Forti* | Diabetes and Hypertension Center of Ceará | adrianaforti@uol.com.br |
| Angela Delmira Nunes Mendes | Diabetes and Hypertension Center of Ceará | angeladelmira@terra.com.br |
| Renan Montenegro Junior* | Federal University of Ceará | renanjr@ufc.br |
| Virgínia Oliveira Fernandes | Federal University of Ceará | virginiafernande@hotmail.com |
| João Soares Felício* | Federal University Hospital of Pará | felicio.bel@terra.com.br |
| Flavia Marques Santos | Federal University Hospital of Pará | [drafms@bol.com.br](mailto:drafms@bol.com.br) |
